# 1-Eth­oxy-2-meth­oxy-4-[2-(4-nitro­phen­yl)ethen­yl]benzene

**DOI:** 10.1107/S1600536812034320

**Published:** 2012-08-25

**Authors:** Paul M. Dinakaran, S. Kalainathan, T. Srinivasan, D. Velmurugan

**Affiliations:** a Crystal Research Centre, School of Advanced Sciences, VIT University, Vellore 632 014, Tamil Nadu, India; b Centre of Advanced Study in Crystallography and Biophysics, University of Madras, Guindy Campus, Chennai 600 025, India

## Abstract

In the title mol­ecule, C_17_H_17_NO_4_, the dihedral angle between the two aromatic rings is 42.47 (7)°. The nitro group is twisted by 7.44 (11)° out of the plane of the ring to which it is attached. The methoxy and ethoxy group O atoms deviate significantly from the phenyl ring [by 0.0108 (11) and 0.0449 (11) Å, respectively]. The crystal structure is stabilized by C—H⋯π inter­actions.

## Related literature

For the synthesis of the title compound, see: Tam *et al.* (1989 ▶). For hybridization, see: Beddoes *et al*. (1986 ▶)
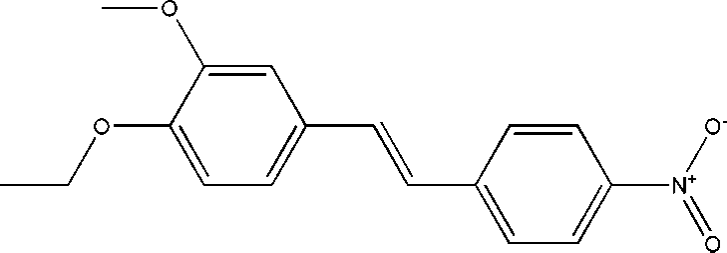


## Experimental

### 

#### Crystal data

C_17_H_17_NO_4_
*M* *_r_* = 299.32
Monoclinic, 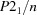

*a* = 8.5209 (4) Å
*b* = 7.5959 (4) Å
*c* = 23.7877 (13) Å
β = 99.611 (3)°
*V* = 1518.02 (14) Å^3^
*Z* = 4
Mo *K*α radiation
μ = 0.09 mm^−1^
*T* = 293 K
0.20 × 0.20 × 0.20 mm

#### Data collection

Bruker SMART APEXII area-detector diffractometer
14265 measured reflections
3789 independent reflections
2831 reflections with *I* > 2σ(*I*)
*R*_int_ = 0.031

#### Refinement

*R*[*F* ^2^ > 2σ(*F* ^2^)] = 0.045
*wR*(*F* ^2^) = 0.138
*S* = 1.04
3789 reflections
202 parameters
H-atom parameters constrained
Δρ_max_ = 0.22 e Å^−3^
Δρ_min_ = −0.19 e Å^−3^

### 

Data collection: *APEX2* (Bruker, 2008 ▶); cell refinement: *SAINT* (Bruker, 2008 ▶); data reduction: *SAINT*; program(s) used to solve structure: *SHELXS97* (Sheldrick, 2008 ▶); program(s) used to refine structure: *SHELXL97* (Sheldrick, 2008 ▶); molecular graphics: *ORTEP-3* (Farrugia, 1997 ▶); software used to prepare material for publication: *SHELXL97* and *PLATON* (Spek, 2009 ▶).

## Supplementary Material

Crystal structure: contains datablock(s) global, I. DOI: 10.1107/S1600536812034320/bt5986sup1.cif

Structure factors: contains datablock(s) I. DOI: 10.1107/S1600536812034320/bt5986Isup2.hkl

Supplementary material file. DOI: 10.1107/S1600536812034320/bt5986Isup3.cml

Additional supplementary materials:  crystallographic information; 3D view; checkCIF report

## Figures and Tables

**Table 1:** Hydrogen-bond geometry (Å, °) *Cg*2 is the centroid of the C9–C14 benzene ring.

*D*—H⋯*A*	*D*—H	H⋯*A*	*D*⋯*A*	*D*—H⋯*A*
C17—H17*A*⋯*Cg*2	0.97	2.96	3.281 (2)	145

**Fig. 1.**
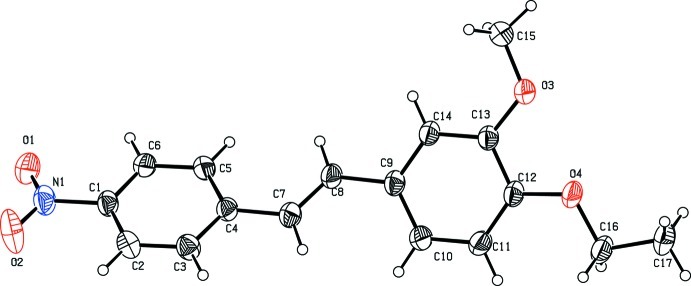
The molecular structure of the title compound, showing displacement ellipsoids drawn at the 30% probability level. H atoms are presented as small spheres of arbitrary radius.

**Fig. 2.**
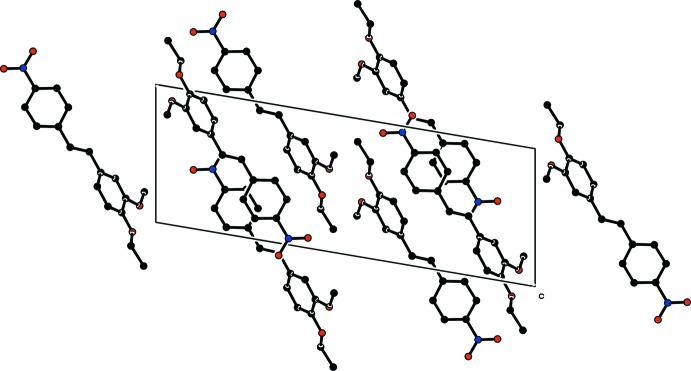
A view of the C—H···N and C—H···O hydrogen bonds (dotted lines) in the crystal structure of the title compound. [Symmetry codes: (i) - x + 1, - y -1, - z; (ii) - x +1/2, y - 1/2, - z + 1/2.]

## supplementary crystallographic information

### Comment

The dihedral angle between nitro substituted phenyl ring (C1-C6) & oxygen substituted benzene ring (C9-C14) is 42.47 (7)°. The sum of bond angles around N1 (359.59°) is in accordance with sp_2_ hybridization (Beddoes *et al.*, 1986). The methoxy and ethoxy group O3 and O4 atoms are significantly deviated from the phenyl ring (C9–C13) with the values of -0.0108 (11) Å and -0.0449 (11) Å, respectively.

A weak intermolecular C—H···π interaction involving the C17–H17A group and the C9–C14 benzene ring (centroid Cg2) of the molecule at (2-x,-y,-z) is observed [H17A···Cg2 = 2.96 Å, C17···Cg2 = 3.781 (2) Å and C17-H17A···Cg2 = 145°].

### Experimental

4-Ethoxy 3-Methoxy 4-Nitrostilbene (4E3MONS) is a derivative material of stilebene.The material (4E3MONS) was synthesized by Witting reaction method. The material was prepared from the 4-ethoxy 3-methoxy benzaldehyde and diethyl p-nitrobenzyl phosphonate in the presence of sodium ethoxide catalyst. The steps involved during the chemical reactions are as follows: the calculated amount of diethyl p-nitrobenzyl phosphonate (0.01 mol %, 2.2304ml) and 4-ethoxy 3-methoxy benzaldehyde (0.01 mol, 1.802 ml %) were added in the ethanol solution (35 ml). After the reaction process, the sodium ethoxide,which plays a role of catalyst, was added immediately the colour of the solution became red. Then the mixture was stirred for 12 hrs at ice cold temperature in ultracryostat which has stirrer rotation facility. After the stirring process completed, the orange colour 4E3MONS material was collected from the mixture by removing the ethanol (Tam *et al.*, 1989). Then the 4E3MONS was purified by a successive recrystallization process.

### Refinement

Hydrogen atoms were placed in calculated positions with C—H = 0.93 Å and refined in riding model with fixed isotropic displacement parameter: *U*_iso_(H) = 1.2 *U*_eq_(C).

### Crystal data

**Table:**

C_17_H_17_NO_4_	*F*(000) = 632
*M**_r_* = 299.32	*D*_x_ = 1.310 Mg m^−^^3^
Monoclinic, *P*2_1_/*n*	Mo *K*α radiation, λ = 0.71073 Å
Hall symbol: -P 2yn	Cell parameters from 3789 reflections
*a* = 8.5209 (4) Å	θ = 1.7–28.5°
*b* = 7.5959 (4) Å	µ = 0.09 mm^−^^1^
*c* = 23.7877 (13) Å	*T* = 293 K
β = 99.611 (3)°	Block, colourless
*V* = 1518.02 (14) Å^3^	0.20 × 0.20 × 0.20 mm
*Z* = 4	

### Data collection

**Table:**

Bruker SMART APEXII area-detector diffractometer	2831 reflections with *I* > 2σ(*I*)
Radiation source: fine-focus sealed tube	*R*_int_ = 0.031
Graphite monochromator	θ_max_ = 28.5°, θ_min_ = 1.7°
ω and φ scans	*h* = −11→11
14265 measured reflections	*k* = −9→9
3789 independent reflections	*l* = −31→30

### Refinement

**Table:**

Refinement on *F*^2^	Secondary atom site location: difference Fourier map
Least-squares matrix: full	Hydrogen site location: inferred from neighbouring sites
*R*[*F*^2^ > 2σ(*F*^2^)] = 0.045	H-atom parameters constrained
*wR*(*F*^2^) = 0.138	*w* = 1/[σ^2^(*F*_o_^2^) + (0.0628*P*)^2^ + 0.2742*P*] where *P* = (*F*_o_^2^ + 2*F*_c_^2^)/3
*S* = 1.04	(Δ/σ)_max_ = 0.001
3789 reflections	Δρ_max_ = 0.22 e Å^−^^3^
202 parameters	Δρ_min_ = −0.19 e Å^−^^3^
0 restraints	Extinction correction: *SHELXL97* (Sheldrick, 2008), Fc^*^=kFc[1+0.001xFc^2^λ^3^/sin(2θ)]^-1/4^
Primary atom site location: structure-invariant direct methods	Extinction coefficient: 0.121 (6)

### Special details

**Table:**

Geometry. All esds (except the esd in the dihedral angle between two l.s. planes) are estimated using the full covariance matrix. The cell esds are taken into account individually in the estimation of esds in distances, angles and torsion angles; correlations between esds in cell parameters are only used when they are defined by crystal symmetry. An approximate (isotropic) treatment of cell esds is used for estimating esds involving l.s. planes.
Refinement. Refinement of F^2^ against ALL reflections. The weighted R-factor wR and goodness of fit S are based on F^2^, conventional R-factors R are based on F, with F set to zero for negative F^2^. The threshold expression of F^2^ > 2sigma(F^2^) is used only for calculating R-factors(gt) etc. and is not relevant to the choice of reflections for refinement. R-factors based on F^2^ are statistically about twice as large as those based on F, and R- factors based on ALL data will be even larger.

### Fractional atomic coordinates and isotropic or equivalent isotropic displacement parameters (Å^2^)

**Table:**

	*x*	*y*	*z*	*U*_iso_*/*U*_eq_	
O4	1.09886 (11)	0.13016 (14)	0.06184 (5)	0.0622 (3)	
O3	0.89997 (12)	0.38803 (13)	0.04273 (5)	0.0619 (3)	
C9	0.70663 (15)	0.13692 (18)	0.14525 (6)	0.0488 (3)	
C13	0.86351 (14)	0.26322 (17)	0.07949 (5)	0.0468 (3)	
C4	0.42645 (15)	0.09455 (17)	0.25675 (6)	0.0469 (3)	
N1	0.04474 (16)	0.11093 (17)	0.34984 (6)	0.0644 (4)	
C1	0.17789 (16)	0.10582 (17)	0.31788 (6)	0.0508 (3)	
C8	0.57030 (15)	0.15205 (19)	0.17555 (6)	0.0512 (3)	
H8	0.4829	0.2161	0.1578	0.061*	
C14	0.73211 (14)	0.26748 (18)	0.10663 (5)	0.0478 (3)	
H14	0.6594	0.3592	0.0990	0.057*	
C6	0.14936 (16)	0.14921 (19)	0.26099 (6)	0.0545 (3)	
H6	0.0478	0.1812	0.2432	0.065*	
C10	0.81278 (17)	−0.0027 (2)	0.15429 (7)	0.0584 (4)	
H10	0.7962	−0.0925	0.1792	0.070*	
C5	0.27400 (16)	0.14451 (19)	0.23074 (6)	0.0529 (3)	
H5	0.2561	0.1752	0.1924	0.063*	
C12	0.97140 (15)	0.12289 (18)	0.08991 (6)	0.0496 (3)	
C11	0.94367 (16)	−0.0096 (2)	0.12641 (7)	0.0583 (4)	
H11	1.0131	−0.1048	0.1325	0.070*	
C7	0.56122 (15)	0.08254 (19)	0.22582 (6)	0.0538 (3)	
H7	0.6494	0.0195	0.2434	0.065*	
O1	−0.08925 (15)	0.13496 (19)	0.32390 (7)	0.0886 (4)	
C3	0.44945 (16)	0.0507 (2)	0.31410 (6)	0.0556 (3)	
H3	0.5502	0.0165	0.3321	0.067*	
C16	1.20153 (17)	−0.0197 (2)	0.06557 (7)	0.0630 (4)	
H16A	1.1425	−0.1227	0.0499	0.076*	
H16B	1.2462	−0.0435	0.1051	0.076*	
C2	0.32634 (17)	0.0564 (2)	0.34522 (6)	0.0579 (4)	
H2	0.3435	0.0274	0.3837	0.070*	
C17	1.33198 (18)	0.0214 (3)	0.03219 (8)	0.0733 (5)	
H17A	1.2867	0.0416	−0.0070	0.110*	
H17B	1.4046	−0.0759	0.0347	0.110*	
H17C	1.3880	0.1250	0.0476	0.110*	
C15	0.7940 (2)	0.5327 (2)	0.03028 (7)	0.0693 (4)	
H15A	0.6912	0.4905	0.0128	0.104*	
H15B	0.8345	0.6119	0.0047	0.104*	
H15C	0.7848	0.5935	0.0650	0.104*	
O2	0.07343 (17)	0.0848 (2)	0.40088 (6)	0.1057 (6)	

### Atomic displacement parameters (Å^2^)

**Table:**

	*U*^11^	*U*^22^	*U*^33^	*U*^12^	*U*^13^	*U*^23^
O4	0.0506 (5)	0.0632 (6)	0.0811 (7)	0.0073 (4)	0.0349 (5)	0.0034 (5)
O3	0.0615 (6)	0.0597 (6)	0.0727 (7)	0.0083 (5)	0.0347 (5)	0.0121 (5)
C9	0.0411 (6)	0.0529 (7)	0.0553 (7)	−0.0035 (5)	0.0169 (5)	−0.0032 (6)
C13	0.0448 (6)	0.0474 (7)	0.0511 (7)	−0.0029 (5)	0.0167 (5)	−0.0033 (5)
C4	0.0443 (6)	0.0456 (7)	0.0534 (7)	−0.0031 (5)	0.0155 (5)	0.0005 (5)
N1	0.0640 (8)	0.0611 (8)	0.0759 (9)	−0.0142 (6)	0.0349 (7)	−0.0055 (6)
C1	0.0525 (7)	0.0461 (7)	0.0592 (8)	−0.0081 (5)	0.0247 (6)	−0.0038 (6)
C8	0.0429 (6)	0.0531 (7)	0.0611 (8)	0.0003 (5)	0.0190 (6)	0.0011 (6)
C14	0.0426 (6)	0.0496 (7)	0.0540 (7)	0.0011 (5)	0.0159 (5)	−0.0040 (6)
C6	0.0465 (7)	0.0549 (8)	0.0647 (9)	0.0044 (6)	0.0169 (6)	0.0081 (6)
C10	0.0531 (7)	0.0541 (8)	0.0730 (10)	0.0013 (6)	0.0252 (7)	0.0095 (7)
C5	0.0496 (7)	0.0595 (8)	0.0522 (7)	0.0033 (6)	0.0157 (6)	0.0090 (6)
C12	0.0411 (6)	0.0530 (7)	0.0586 (8)	−0.0017 (5)	0.0195 (5)	−0.0055 (6)
C11	0.0496 (7)	0.0528 (8)	0.0770 (10)	0.0071 (6)	0.0234 (7)	0.0046 (7)
C7	0.0420 (6)	0.0619 (8)	0.0594 (8)	0.0016 (6)	0.0145 (6)	0.0035 (7)
O1	0.0603 (7)	0.1011 (10)	0.1138 (10)	0.0131 (7)	0.0422 (7)	0.0163 (8)
C3	0.0479 (7)	0.0656 (9)	0.0531 (8)	−0.0037 (6)	0.0082 (6)	0.0046 (7)
C16	0.0515 (7)	0.0690 (10)	0.0734 (10)	0.0109 (7)	0.0250 (7)	−0.0059 (8)
C2	0.0604 (8)	0.0668 (9)	0.0485 (7)	−0.0102 (7)	0.0143 (6)	0.0016 (6)
C17	0.0513 (8)	0.0977 (13)	0.0768 (11)	0.0075 (8)	0.0276 (7)	−0.0102 (9)
C15	0.0742 (10)	0.0692 (10)	0.0708 (10)	0.0166 (8)	0.0301 (8)	0.0191 (8)
O2	0.0873 (9)	0.1721 (16)	0.0668 (8)	−0.0343 (10)	0.0395 (7)	−0.0100 (9)

### Geometric parameters (Å, º)

**Table:**

O4—C12	1.3678 (14)	C6—C5	1.3791 (18)
O4—C16	1.4296 (17)	C6—H6	0.9300
O3—C13	1.3605 (16)	C10—C11	1.3905 (18)
O3—C15	1.4218 (18)	C10—H10	0.9300
C9—C10	1.3871 (19)	C5—H5	0.9300
C9—C14	1.3933 (19)	C12—C11	1.375 (2)
C9—C8	1.4702 (17)	C11—H11	0.9300
C13—C14	1.3831 (16)	C7—H7	0.9300
C13—C12	1.4024 (18)	C3—C2	1.3818 (19)
C4—C3	1.386 (2)	C3—H3	0.9300
C4—C5	1.3950 (18)	C16—C17	1.503 (2)
C4—C7	1.4664 (18)	C16—H16A	0.9700
N1—O2	1.2141 (19)	C16—H16B	0.9700
N1—O1	1.2175 (19)	C2—H2	0.9300
N1—C1	1.4678 (17)	C17—H17A	0.9600
C1—C2	1.375 (2)	C17—H17B	0.9600
C1—C6	1.375 (2)	C17—H17C	0.9600
C8—C7	1.321 (2)	C15—H15A	0.9600
C8—H8	0.9300	C15—H15B	0.9600
C14—H14	0.9300	C15—H15C	0.9600
			
C12—O4—C16	117.72 (11)	O4—C12—C13	115.69 (12)
C13—O3—C15	117.87 (10)	C11—C12—C13	119.43 (11)
C10—C9—C14	118.49 (12)	C12—C11—C10	120.62 (13)
C10—C9—C8	122.14 (12)	C12—C11—H11	119.7
C14—C9—C8	119.37 (12)	C10—C11—H11	119.7
O3—C13—C14	124.98 (12)	C8—C7—C4	126.75 (13)
O3—C13—C12	115.47 (11)	C8—C7—H7	116.6
C14—C13—C12	119.54 (12)	C4—C7—H7	116.6
C3—C4—C5	118.04 (12)	C2—C3—C4	121.63 (13)
C3—C4—C7	119.01 (12)	C2—C3—H3	119.2
C5—C4—C7	122.94 (12)	C4—C3—H3	119.2
O2—N1—O1	123.13 (14)	O4—C16—C17	107.48 (14)
O2—N1—C1	118.00 (14)	O4—C16—H16A	110.2
O1—N1—C1	118.82 (14)	C17—C16—H16A	110.2
C2—C1—C6	121.92 (12)	O4—C16—H16B	110.2
C2—C1—N1	119.48 (13)	C17—C16—H16B	110.2
C6—C1—N1	118.59 (13)	H16A—C16—H16B	108.5
C7—C8—C9	125.65 (13)	C1—C2—C3	118.44 (13)
C7—C8—H8	117.2	C1—C2—H2	120.8
C9—C8—H8	117.2	C3—C2—H2	120.8
C13—C14—C9	121.24 (12)	C16—C17—H17A	109.5
C13—C14—H14	119.4	C16—C17—H17B	109.5
C9—C14—H14	119.4	H17A—C17—H17B	109.5
C1—C6—C5	118.84 (13)	C16—C17—H17C	109.5
C1—C6—H6	120.6	H17A—C17—H17C	109.5
C5—C6—H6	120.6	H17B—C17—H17C	109.5
C9—C10—C11	120.61 (13)	O3—C15—H15A	109.5
C9—C10—H10	119.7	O3—C15—H15B	109.5
C11—C10—H10	119.7	H15A—C15—H15B	109.5
C6—C5—C4	121.13 (13)	O3—C15—H15C	109.5
C6—C5—H5	119.4	H15A—C15—H15C	109.5
C4—C5—H5	119.4	H15B—C15—H15C	109.5
O4—C12—C11	124.88 (12)		
			
C15—O3—C13—C14	−1.2 (2)	C16—O4—C12—C11	7.3 (2)
C15—O3—C13—C12	179.55 (13)	C16—O4—C12—C13	−172.56 (13)
O2—N1—C1—C2	5.9 (2)	O3—C13—C12—O4	0.07 (18)
O1—N1—C1—C2	−171.61 (15)	C14—C13—C12—O4	−179.27 (12)
O2—N1—C1—C6	−174.80 (15)	O3—C13—C12—C11	−179.80 (13)
O1—N1—C1—C6	7.7 (2)	C14—C13—C12—C11	0.9 (2)
C10—C9—C8—C7	25.6 (2)	O4—C12—C11—C10	178.12 (14)
C14—C9—C8—C7	−153.64 (15)	C13—C12—C11—C10	−2.0 (2)
O3—C13—C14—C9	−177.74 (13)	C9—C10—C11—C12	0.8 (2)
C12—C13—C14—C9	1.5 (2)	C9—C8—C7—C4	−179.53 (13)
C10—C9—C14—C13	−2.7 (2)	C3—C4—C7—C8	−164.96 (15)
C8—C9—C14—C13	176.55 (12)	C5—C4—C7—C8	16.6 (2)
C2—C1—C6—C5	−0.7 (2)	C5—C4—C3—C2	−0.3 (2)
N1—C1—C6—C5	−179.95 (13)	C7—C4—C3—C2	−178.83 (13)
C14—C9—C10—C11	1.5 (2)	C12—O4—C16—C17	−179.27 (13)
C8—C9—C10—C11	−177.69 (14)	C6—C1—C2—C3	0.0 (2)
C1—C6—C5—C4	0.9 (2)	N1—C1—C2—C3	179.29 (13)
C3—C4—C5—C6	−0.4 (2)	C4—C3—C2—C1	0.5 (2)
C7—C4—C5—C6	178.09 (13)		

### Hydrogen-bond geometry (Å, º)

Cg2 is the centroid of the C9–C14 benzene ring.

**Table:**

*D*—H···*A*	*D*—H	H···*A*	*D*···*A*	*D*—H···*A*
C17—H17*A*···*Cg*2	0.97	2.96	3.281 (2)	145
